# The effect of home-based exercise on motor symptoms, quality of life and functional performance in Parkinson's disease: a systematic review and meta-analysis

**DOI:** 10.1186/s12877-023-04595-6

**Published:** 2023-12-19

**Authors:** Yong Yang, Xueying Fu, Haoyang Zhang, Guoguang Ouyang, Shu-Cheng Lin

**Affiliations:** 1 Laboratory of Kinesiology and Rehabilitation, School of Physical Education and Sport, Chaohu University, Hefei, 238000 China; 2 Laboratory of Kinesiology and Rehabilitation, School of Physical Education and Sport, Henan University, Kaifeng, 475001 China; 3 Nuanquan Middle School, Yingkou, China; 4 Department of Sport, Leisure and Health Management, Tainan University of Technology, Tainan City, 710302 Taiwan

**Keywords:** Home-based exercise, COVID-19, Physical activity, Parkinson's disease patients, Motor symptoms

## Abstract

**Background:**

Faced with the lack of physical activity caused by mandatory home isolation during special periods and patients' inconvenience in carrying out professionally supervised exercise, many home-based exercise programs have been developed. This systematic review and meta-analysis aimed to examine the effects of home-based exercise on measures of motor symptoms, quality of life and functional performance in Parkinson's disease (PD) patients.

**Methods:**

We performed a systematic review and meta-analysis, and searched PubMed, MEDLINE, Embase, Cochrane library, and Web of Science from their inception date to April 1, 2023. The quality of the literature was assessed using PEDro's quality scale. The data was pooled using R software. Results are presented as pooled standardized mean difference (SMD) with 95% confidence interval (CI).

**Results:**

A total of 20 studies involving 1885 PD patients were included. Meta-analysis results showed that home-based exercise had a small effect in relieving overall motor symptoms in PD patients (SMD = -0.29 [-0.45, -0.13]; *P* < 0.0001), improving quality of life (SMD = 0.20 [0.08, 0.32]; *P* < 0.0001), walking speed (SMD = 0.26 [0.05, 0.48]; *P* = 0.005), balance ability (SMD = 0.23 [0.10, 0.36]; *P* < 0.0001), finger dexterity (SMD = 0.28 [0.10, 0.46]; *P* = 0.003) and decreasing fear of falling (SMD = -0.29 [-0.49, -0.08]; *P* = 0.001). However, home-based exercise did not significantly relieve the overall motor symptoms of PD patients when the training period was less than 8 weeks and the total number of sessions was less than 30.

**Conclusion:**

During times of limited physical activity due to pandemics such as COVID-19, home-based exercise is an alternative to maintain and improve motor symptoms in PD patients. In addition, for the minimum dose of home-based exercise, we recommend that the exercise period is no less than 8 weeks and the total number of sessions is no less than 30 times.

**Trial registration:**

PROSPERO registration number: CRD42022329780.

## Contribution of the paper

1. Home based exercise had a small effect in relieving overall motor symptoms in PD patients, improving quality of life, walking speed, balance ability, finger dexterity, and decreasing fear of falling.
2. In terms of exercise dosage, we recommend the exercise period is no less than 8 weeks and the total number of sessions is no less than 30 times.

## Introduction

Severe acute respiratory syndrome coronavirus causes coronavirus disease 2019 (COVID-19) and has triggered a pandemic with serious medical conditions, including death, economic disruption, and deterioration of the health of the virus-free population due to mandatory self-isolation. Long-term home isolation can significantly increase physical inactivity. China conducted a nationwide cross-sectional study in the early days of the COVID-19 outbreak, using an online questionnaire and collecting 7-day physical activity, sedentary screen time, and emotional state. Findings from 12,107 participants aged 18–80 indicated that nearly 60% of older adults were not achieving the amount of physical activity required for health benefits. In the non-pandemic period, the proportion was only 14% [1].

Research in the Journal of Parkinson’s Disease [2] suggested that the COVID-19 pandemic has led to worsened symptoms by evoking psychological distress and reducing physical activity—an important component of many Parkinson disease (PD) patients treatment plans [3]. Additionally, a scoping review in Public Health indicated that because of the pandemic, individuals with PD worldwide reported decreased physical and mental health, daily activities, and social support, as well as discontinuation of regular health care and physical therapy appointments [4]. The results of 5,429 surveys from Parkinson's patients analyzed by Fox Insight, the MJFF's online clinical study, showed that they experienced worsening of motor and non-motor symptoms regardless of whether they were diagnosed with COVID-19. They also reported disruptions in exercise, social activity and healthcare for PD patients [5].

Numerous research findings support the positive effects of exercise and physical activity on PD patients [6–8]. During the ongoing COVID-19 pandemic, a home-based exercise program constitutes a viable strategy for relieving the exacerbation of motor symptoms associated with inactivity in PD patients [9–11]. In addition to recent calls to stay physically active even when forced to isolate at home due to the COVID-19 crisis [12, 13]. World Health Organization (WHO) has also launched a "Maintain Physical Activity at Home During COVID-19" campaign to urge people to maintain their daily physical activity. However, the WHO recommendation does not specify the type and dose of exercise.

Experimental evidence supports the beneficial effects of home-based exercise on motor symptoms in PD patients. However, different types and doses of exercise lead to different effects in slowing the progression of PD patients. van der Kolk, et al. (2019) [11] conducted a total of 72 sessions of high-intensity aerobic exercise for 24 weeks on 130 PD patients, and the results showed that aerobic exercise significantly alleviated the motor symptoms of PD patients (reflected in Unified Parkinson's Disease Rating Scale motor, (UPDRS-motor)). When the aerobic exercise period was reduced to 6 weeks (24 sessions), no effective relief was found [14]. When aerobic and resistance training were combined, engaging twice a week for 24 weeks, there was also an effective alleviation of motor symptoms [15]. This evidence has not been comprehensively and systematically evaluated. Therefore, this systematic review and meta-analysis aimed to comprehensively and systematically assess the impact of home-based exercise on motor symptoms and functional performance in patients with PD, and to identify effective exercise types and doses through subgroup analysis. Our study offers practical recommendations for individuals with PD who find themselves in situations where they must self-isolate at home due to COVID-19 or have limited mobility, preventing them from participating in supervised exercise programs. Meanwhile, we suggest broadening the scope to emphasize the potential benefits of additional home-based training for all PD patients as a supplementary approach to conventional therapy.

## Methods

This systematic review was performed according to the Cochrane group [16] and according to the Preferred Reporting Items for Systematic Reviews and Meta-Analyses (PRISMA) statement [17]. The review protocol was registered with PROSPERO (CRD42022329780).

### Search strategy and selection criteria

From February 23, 2023 to April 1, 2023, two investigators independently searched for eligible studies, a systematic literature search covering the period from inception to April 1, 2023 was performed using various electronic databases: PubMed, Cochrane, Embase, Medline, and Web of Science. The following terms were used to perform the electronic searches: “exercises” OR “physical therapy” OR “physical activity”) AND (“Parkinson’s Disease” OR “Parkinson” OR “PD”) AND (“home-base” OR “remotely supervised”) AND ((random* OR control*). A manual search was also performed in the reference list of included articles and previously published reviews, in order to retrieve articles not covered by the databases search.

### Eligibility criteria

In accordance with the PICOS approach [17], the inclusion criteria were as follows: (a) participants: Individuals with PD, the mean age ≥ 50 years, Hoehn and Yahr stages < 4; (b) intervention: home-base self-supervised or remotely supervised exercises (e.g. aerobic exercise, resistance training, balance training, or a combination of the above forms of exercise); (c) comparator: non-physically active (e.g., health education, no activity intervention, and usual care) control groups; (d) outcomes: the primary outcome was the changes in total motor symptoms of PD as measured by the UPDRS-motor. The secondary outcomes were quality of life (e.g., the Parkinson's Disease Questionnaire (PDQ-39). In addition, we also assessed balance ability (e.g., berg balance scale (BBS)), walking speed for 10 m walking test, fear of falling for fall efficacy scale–international questionnaire (FES-I) and finger dexterity for nine-hole peg test; (e) study design: included RCTs of individual-designed, cluster-designed, or the first half of crossover. Exclusion criteria were: (a) single-session interventions; (b) studies examining the effects of nutritional supplements in combination with exercises; (c) abstract and conference proceedings.

### Study selection and data extraction

The final selected articles entered into the meta-analysis process were prepared to be extracted using a preprepared checklist. The checklist includes demographic characteristics, sample size, H&Y stage, duration of disease, ON/OFF state of outcomes assessed, intervention type (experimental and control), intervention characteristics (periods, frequency, intensity, and total time per session), and pre- and post-intervention results (expressed as mean ± standard deviation [SD] when available).

### Risk of bias

The risk of bias for each individual study was assessed independently by XYF and HYZ using the Physiotherapy Evidence Database (PEDro) scale, and interrater reliability was shown to be fair to good (intraclass correlation coefficient = 0.68) [18]. The PEDro scale scores internal validity of the studies and the presence of statistically replicable information on a scale of 0 (high risk of bias) to 10 (low risk of bias), and ≥ 6 represents the cut-off score for studies with low risk of bias.

### Statistical analyses

The pooled effect of exercise on PD was estimated through a random-effects model meta-analysis [19] when at least two studies used the same outcome. “R” software packages “meta” were used in this article for data analysis. The standardised mean difference (SMD) and 95% CI were used for pooling effects. The SMDs were interpreted using the conventions as outlined by Cohen [20] (SMD < 0.2 “trivial”; 0.2 ≤ SMD < 0.5 “small”, 0.5 ≤ SMD < 0.8 “moderate”, SMD ≥ 0.8 “large”). Subgroup analyses attending to the different exercise modalities and doses (exercise period, frequency, total number of courses, and weekly exercise time) were performed for the total motor symptoms. Regarding the exercise modalities, we divided it into mix exercise and aerobic exercise; exercise period was ≤ 8, > 8–16, and > 16 weeks; frequency was ≤ 3 and > 3; total number of curses was ≤ 30, > 30–60, and > 60; and weekly exercise time was ≤ 90–120 and > 120 min according to the characteristics of the included studies. The weight assigned to each study included in the meta-analysis was defined by the SD of the variables and the sample size. Statistical heterogeneity among the studies was assessed by I^2^ value. The synthesis of included trials was considered as a significant heterogeneity if I^2^ > 50% [21]. All statistical significance levels were set at *p* < 0.05.

## Results

### Study selection

**Fig. 1:**
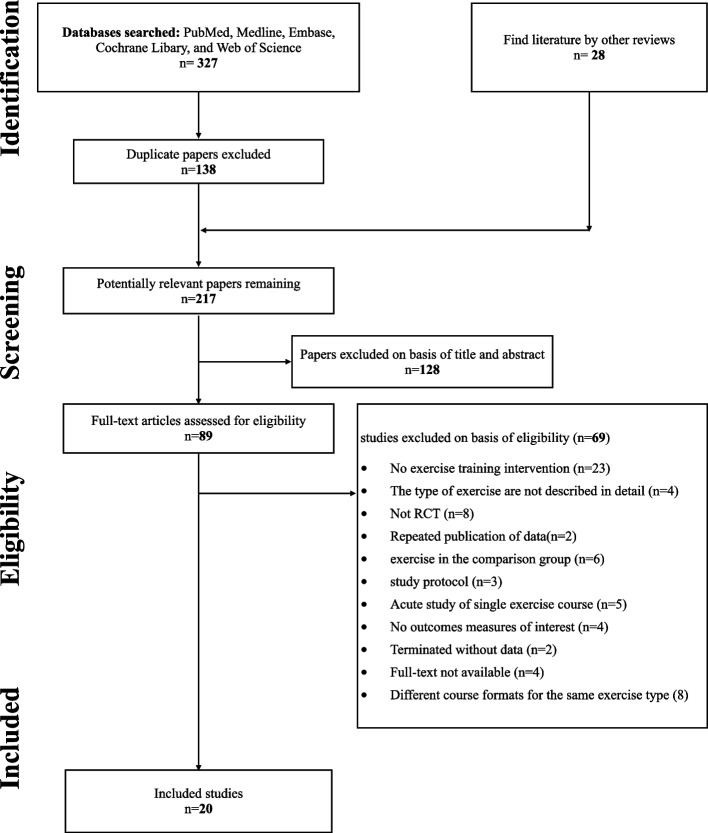
Flow chart of literature search and study selection

The literature search yielded 327 articles. After removal of duplicates, 217 studies were screened on the basis of the title and abstract, of these, 128 studies did not meet the inclusion criteria. A total of 89 unique full-text articles were assessed for eligibility. At the end of the screening phase, 20 studies (1885 subjects) were included in systematic review (Fig. 1).

### Studies characteristics

A comprehensive summary of the trials and participants’ characteristics is reported in the Table 1. All the 20 studies included were randomized, one [22] (5.0%) was crossover, and 19 (95.0%) had a parallel design. The sample size per study was 94.3 ± 97.8 (mean ± SD) with a total of 1885 PD participants (age: 66.3 ± 5.3, years; disease duration: 7.3 ± 2.5, years; H & Y: 2.3 ± 0.5, stage). Type of treatment in the 20 selected trials was varied: aerobic exercise (*n* = 5) [9, 11, 14, 23, 24], balance and gait training (*n* = 4) [10, 22, 25, 26], combination of aerobic, strength, or balance exercise (*n* = 10) [15, 27–35], arm and hand exercise (*n* = 1) [36]. Exercise period and total number of intervention sessions for each trial differed significantly among the studies (range, 3–24 weeks, 9–96 sessions; mean ± SD, 12.5 ± 7.8 weeks, 38.1 ± 25.8 sessions), whereas training frequency was quite similar in the most of the studies (range, 2–7; mean ± SD, 3.1 ± 1.3). In 3[9, 29, 35] of the 20 studies, treatment effects were evaluated when patients were in the OFF phase (> 12 h after having taken the medication). In 16 [10, 14, 15, 22–28, 31–34, 36, 37] of the 20 studies, treatment effects were evaluated when patients were in the ON phase (1–2 h after having taken the medication). Only one [11] included study evaluated the treatment effects of both ON and OFF phase. The mean PEDro score of the included studies was 6.8 (median 7), and 18 of the 20 studies achieved the cut-off value of 6 (Table 2).

**Table 1:** The basic characteristics of included studies

Study	Age (mean ± SD)		Sample size (Male/Female)		Disease duration (years, mean ± SD)		H&Y		ON/OFF	P	F	Intensity	Time	Intervention detail		Compliance	Indicators of interest
	**EXP**	**CON**	**EXP**	**CON**	**EXP**	**CON**	**EXP**	**CON**						**EXP**	**CON**		
Allen et al. (2010) [31]	66 ± 10	68 ± 7	24 (13/11)	24 (13/11)	7 ± 5	9 ± 6	NA		ON	24	3	NA	40–60	strength and balance training	usual care	70%	PDQ 39, score; Walking speed, m/s; FES-I, score
Allen et al. (2017) [36]	67.5 ± 7.3	68.5 ± 8.5	19 (12/7)	19 (11/8)	NA		NA		ON	12	3	NA	NA	An interactive videogame for arm and hand exercise	no activity intervention	NA	PDQ 39, score; Nine Hole Peg Board test, s
Ashburn et al. (2007) [27]	72.7 ± 9.6	71.6 ± 8.8	70 (38/32)	72 (48/24)	7.7 ± 5.8	9.0 ± 5.8	3.1 ± 0.8	3.0 ± 0.7	ON	24		NA	60	strength, aerobic, balance and gait training	usual care	94%	BBS, score
Caglar et al. (2005) [28]	67.4 ± 5.04	64.3 ± 12.3	15 (11/4)	15 (10/5)	5.5 ± 2.7	5.2 ± 2.7	2.1 ± 0.50	2.1 ± 0.57	ON	8	7	NA	60	aerobic exercise, resistance training, balance training	routine activities	NA	Nine Hole Peg Board test, s
Canning et al. (2012) [14]	60.7 ± 5.9	62.9 ± 9.9	10 (5/5)	10 (6/4)	6.1 ± 4.0	5.2 ± 4.1	I–II		ON	6	4	50–80% 6 MWT speed	30–40	treadmill training	no activity intervention	78%	UPDRS-III, score; PDQ 39, score; 10 m walking test, m/s;
Collett et al. (2017) [15]	66 ± 9	67 ± 7	54 (31/23)	51 (30/21)	4.8 ± 4.1	5.3 ± 4.1	NA		ON	24	2	55–85% HRmax	60	resistance training, and aerobic exercise	handwriting	97%	MDS-UPDRS-III, score; Nine Hole Peg Board test, s
Goodwin et al. (2011) [29]	72.0 ± 8.6	70.1 ± 8.3	64 (39/25)	66 (35/31)	9.1 ± 6.4	8.2 ± 6.4	2.6 ± 0.9	2.4 ± 0.9	OFF	10	2	NA	60	resistance training, and balance training	usual care	70%	BBS, score; FES-I, score
Johansson et al. (2022) [9]	58.9 ± 8.9	59.8 ± 10.1	26 (20/6)	31 (17/14)	3.7 ± 3.2	3.96 ± 2.7	NA		OFF	24	3	50–80% HRR	30–45	aerobic exercise based on cycling	relaxation exercises	NA	MDS-UPDRS-III, score;
Khalil et al. (2017) [23]	58.4 ± 13.5	60.7 ± 15.4	16 (12/4)	14 (7/7)	8.0 ± 6.4	7.5 ± 4.0	2.4 ± 0.7	2.2 ± 0.8	ON	8	3	RPE 3–4	45	aerobic exercise	usual care	77%	MDS-UPDRS-III, score; 10 m walking test, m/s; FES-I, score
Khobkhun et al. (2021) [32]	66.5 ± 4.2	67.0 ± 4.4	10 (NA)	10 (NA)	6.4 ± 3.5	6.6 ± 3.9	2.3 ± 0.5	2.3 ± 0.5	ON	10	7	NA	60	strength, balance, and gait exercise	maintain daily activities	NA	MDS-UPDRS-III, score
Morris et al. (2017) [30]	71 ± 8	71 ± 10	67 (45/22)	66 (35/31)	NA		2.3 ± 0.7	2.4 ± 0.7	ON	6	2	NA	60	resistance training, balance training, and aerobic training	health education	92.50%	UPDRS-III, score; PDQ 39, score
Nieuwboer et al. (2014)	67.5 ± 7.8	69 ± 7.8	76 (48/28)	77 (40/37)	7 ± 5.2	8 ± 5.9	2.5 ± 0.4	3 ± 0.4	ON	3	3	NA	30	balance and gait training	no activity intervention	NA	PDQ 39, score; 10 m walking test, m/s; FES-I, score
Seymour et al. (2019) [26]	71 ± 7.7	73 ± 7.7	238 (147/91)	236 (119/117)	8 ± 6.6	8 ± 5.8	2.6 ± 0.4	2.7 ± 0.5	ON	24	3	NA	60	balance and gait training	health education	91%	PDQ 39, score; FES-I, score
Song et al. (2017)	68 ± 7	65 ± 7	31 (15/16)	29 (9/20)	7 ± 4	9 ± 6	NA		ON	12	3	NA	15	step training game	no activity intervention	86%	Gait adaptability test, cm/s; FES-I, score
Stack et al. (2011)^a^	75 ± 5.9	74 ± 5.9	24 (17/7)	23 (18/5)	8 ± 5.2	7 ± 5.9	3.2 ± 0.6	3 ± 0.7	ON	4		NA	NA	strength and gait exercise	usual care	74%	PDQ 39
Tickle-Degnen et al. (2010) [34]	EXP1: 65.8 ± 8.3 EXP2: 67.6 ± 10.3	65.6 ± 8.3	EXP1: 33 (23/10) EXP2: 37 (27/10)	37 (25/12)	EXP1: 7.0 ± 5.3 EXP2: 7.2 ± 6.0	7.0 ± 5.8	EXP1: 2.3 ± 0.5 EXP2: 2.4 ± 0.6	2.3 ± 0.4	ON	6	2	NA	60	EXP1: gait training, strength, stretch EXP2: gait training, strength, stretch, health education	no activity intervention	NA	PDQ 39, score
van der Kolk et al. (2018)^a^ [24]	56.7 ± 5.3	58.4 ± 6.3	22 (NA)	15 (NA)	NA		2.0 ± 0.4	2.1 ± 0.3	ON	24	3	NA	30	aerobic exercise	no activity intervention	92%	UPDRS-III, score; PDQ 39, score; Nine Hole Peg Board test, s
van der Kolk et al. (2019)^a^ [11]	59.3 ± 8.3	59.4 ± 9.3	65 (42/23)	65 (38/27)	15.1 ± 4.0	16.1 ± 4.5	1.9 ± 0.3	2.0 ± 0.2	ON/OFF	24	3	76.4% HRmax	30–45	aerobic exercise	stretch and relaxation	65%	MDS-UPDRS-III, score (ON/OFF); PDQ 39, score; Nine Hole Peg Board test, s
Vasconcellos et al. (2021)^a^	66.0 ± 6.3	65.4 ± 10.1	14 (10/4)	14 (8/6)	6.1 ± 4.4	6.0 ± 4.7	2.7 ± 0.7	2.7 ± 0.6	ON	3	3	NA	60	balance and gait training	stretch	67%	Gait speed, m/s
Xiao et al. (2015)	68.2 ± 2.3	66.5 ± 2.1	48 (33/15)	48 (34/14)	5.5 ± 3.6	6.2 ± 2.6	2.2 ± 0.2	2.1 ± 0.2	OFF	24	4	43–49% HRmax	45	Qigong	no activity intervention	NA	UPDRS-III, score; Gait speed, m/s; BBS, score

*EXP* Experimental group, *CON* Controlled group; OFF: > 12 h after having taken the medication, *SD* Standard deviation ON: 1–2 h after having taken the medication, *H&Y* Hoehn and Yahr stages

^a^telerehabilitation supervised; *P* exercise period, *F* exercise frequency, *UPDRS* III Unified Parkinson's Disease Rating Scale motor symptoms, *PDQ-39* Parkinson's Disease Questionnaire-39, *FES-I* Falls Efficacy Scale – International, *BBS* Berg Balance Scale

**Table 2:** Physiotherapy Evidence Database (PEDro) scores of the 20 included studies

Authors	Eligibility criteria	Random allocation	Concealed allocation	Baseline comparability	Blind subjects	Blind therapists	Blind assessor	Adequate follow-up dropout < 15%	Intention-to- treat analysis	Between-group comparisons	Point estimates and variability	Score
Allen et al. (2010) [31]	1	1	0	1	0	1	0	1	1	1	1	7
Allen et al. (2017) [36]	1	1	1	1	0	1	0	1	1	1	0	7
Ashburn et al. (2007) [27]	1	1	0	1	0	0	1	1	1	1	1	7
Caglar et al. (2005) [28]	1	1	0	1	1	1	1	1	0	1	1	8
Canning et al. (2012) [14]	1	1	0	1	0	0	1	1	1	1	1	7
Chivers Seymour et al. (2019) [26]	1	1	1	1	0	0	1	1	1	1	1	8
Collett et al. (2017) [15]	1	1	1	1	0	0	1	1	1	1	1	8
Goodwin et al. (2011) [29]	1	1	0	1	0	0	0	1	1	1	1	6
Johansson et al. (2022) [9]	1	1	0	1	0	0	0	1	1	1	0	5
Khalil et al. (2017) [23]	1	1	1	1	0	0	1	1	0	1	1	7
Khobkhun et al. (2021) [32]	1	1	0	1	0	0	0	1	1	1	0	5
Morris et al. (2017) [30]	1	1	1	1	0	0	1	1	1	1	0	7
Nieuwboer et al. (2007) [22]	1	1	1	1	0	0	1	1	1	1	1	8
Song et al. (2018) [25]	1	1	0	1	0	0	1	1	1	1	1	7
Stack et al. (2012) [33]	1	1	1	1	0	0	1	1	0	1	0	6
Tickle-Degnen et al. (2010) [34]	1	1	0	1	0	0	0	1	1	1	1	6
van der Kolk et al. (2018) [24]	1	1	0	1	0	0	1	1	0	1	1	6
van der Kolk et al. (2019)	1	1	0	1	1	0	1	1	1	1	1	8
Vasconcellos et al. (2021)	1	1	0	1	0	0	1	1	1	1	1	7
Xiao and Zhuang (2016) [35]	1	1	0	1	0	0	1	1	1	1	0	6
Mean ± SD												6.8 ± 0.95

### Effectiveness of home exercises

**Fig. 2:**
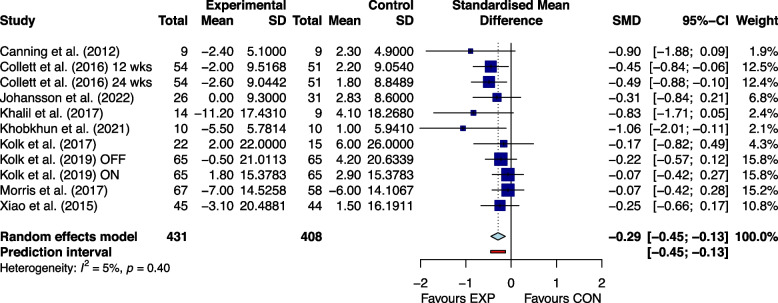
The effect of home-based exercise on overall motor symptoms in Parkinson's disease patients. SMD standard mean deviation, CI confidence interval

**Fig. 3:**
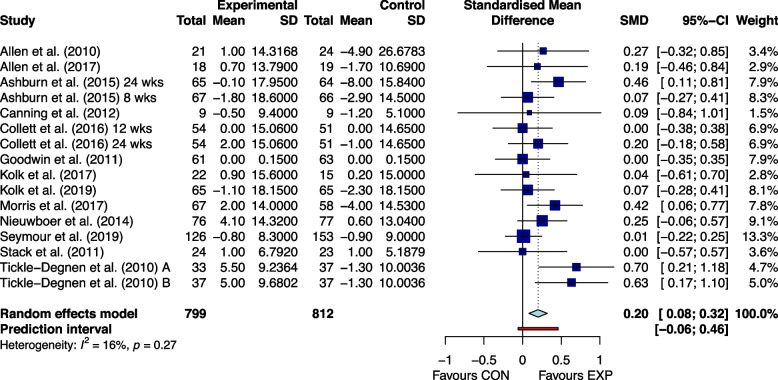
The effect of home-based exercise on quality of life in Parkinson's disease patients. SMD standard mean deviation, CI confidence interval

**Fig. 4:**
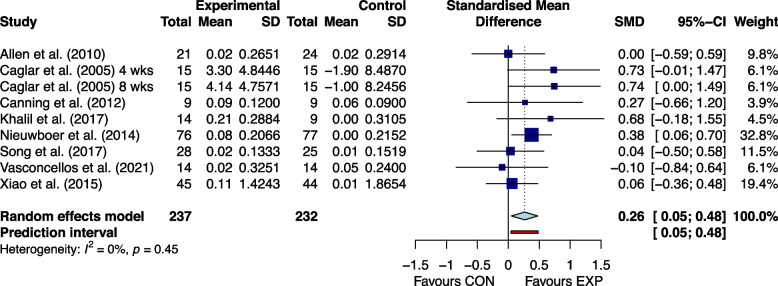
The effect of home-based exercise on walking speed in Parkinson's disease patients. SMD standard mean deviation, CI confidence interval

**Fig. 5:**
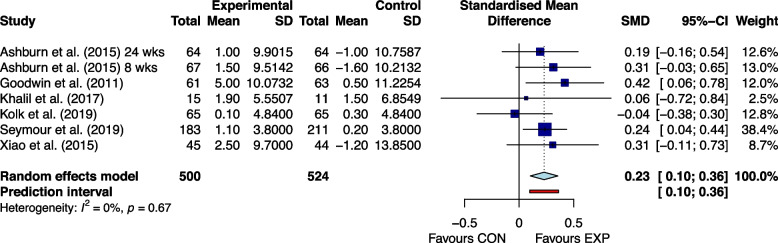
The effect of home-based exercise on balance ability in Parkinson's disease patients. SMD standard mean deviation, CI confidence interval

**Fig. 6:**
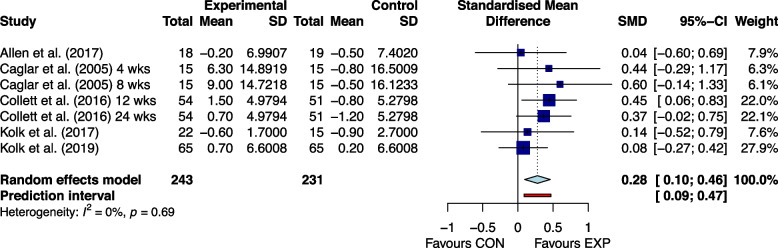
The effect of home-based exercise on finger dexterity in Parkinson's disease patients. SMD standard mean deviation, CI confidence interval

**Fig. 7:**
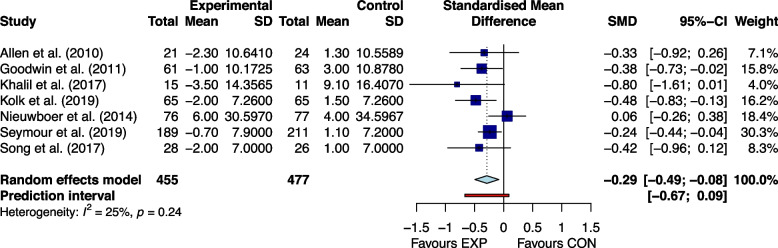
The effect of home-based exercise on fear of falling in Parkinson's disease patients. SMD standard mean deviation, CI confidence interval

Figures 2, 3, 4, 5, 6 and 7 showed the effects of home-based exercise on outcomes related to motor symptoms in PD patients compared with the control group. Home-based exercise had small effects on reducing overall motor symptoms in PD patients (SMD = -0.29 [-0.45,-0.13]; *P* < 0.0001, Fig. 2), improving quality of life (SMD = 0.20 [0.08, 0.32]; P < 0.0001, Fig. 3), walking speed (SMD = 0.26 [0.05, 0.48]; *P* = 0.005, Fig. 4), balance ability (SMD = 0.23 [0.10, 0.36]; *P* < 0.0001, Fig. 5), finger dexterity (SMD = 0.28 [0.10, 0.46]; *P* = 0.003, Fig. 6) and decreased fear of falling (SMD = -0.29 [-0.49, -0.08]; *P* = 0.001, Fig. 7).

### Subgroup analysis results

Table 3 shows the results of the subgroup analysis. Both aerobic and mixed exercise had small effects on overall motor symptom relief in PD patients (aerobic: SMD = -0.29 [-0.45, -0.13], *P* = 0.019, 5 studies; mixed: SMD = -0.35 [ -0.57, -0.12], *P* < 0.0001, 4 studies). However, home-based exercise did not significantly relieve the overall motor symptoms of PD patients when the training period was less than 8 weeks and the total number of sessions was less than 30. It was worth noting that when the training period was 8–16 weeks, the weekly exercise frequency was greater than 3 times, and the weekly exercise time was greater than or equal to 120 min, home-based exercise had a moderate effect on relieving the overall motor symptoms of PD patients.

**Table 3:** Subgroup analysis of exercise types and doses on overall motor symptoms in Parkinson's disease patients

	**Overall motor symptoms**				
	**SMD [95% CI]**	**S (I)**	**N**	**I**^**2**^** (%)**	***p***
Pooling effects	SMD = **-0.29** [-0.45, -0.13]	9 (11)	312	5	< 0.0001
Exercise types					
Aerobic exercise	SMD = **-0.24** [-0.44, -0.04]	5 (6)	136	0	0.019
Mix exercise	SMD = **-0.35** [-0.57, -0.12]	4 (5)	176	28	0.011
Exercise period (weeks)					
≤ 8	SMD = -0.46 [-1.07, 0.14]	3 (3)	90	54	0.133
> 8–16	SMD = **-0.60** [-1.11, -0.08]	2 (2)	64	27	0.023
> 16	SMD = **-0.25** [-0.41, -0.08]	5 (6)	158	0	0.004
Frequency (times/week)					
≤ 3	SMD = **-0.26** [-0.41, -0.11]	6 (8)	248	0	0.001
> 3	SMD = **-0.58** [-1.13, -0.03]	3 (3)	64	39	0.037
Total number of courses					
≤ 30	SMD = -0.46 [-1.07, 0.14]	3 (3)	90	54	0.133
> 30–60	SMD = **-0.47** [-0.74, -0.19]	1 (2)	54	0	0.001
> 60	SMD = **-0.22** [-0.41, -0.04]	5 (6)	168	0	0.018
Weekly exercise time (minutes)					
≤ 90–120	SMD = **-0.24** [-0.39, -0.09]	5 (7)	234	0	0.001
> 120	SMD = **-0.59** [-1.01, -0.17]	4 (4)	78	24	0.006

Bold type represents significant effect

*SMD* Weighted standard mean difference, *CI* Confidence interval, *S (I)* Number of included studies (pooled SMD number), *N* Total number of subjects included in the experimental group of the study

## Discussion

The main findings of this study showed that: 1) Home-based exercise has small effects on relieving the overall motor symptoms, improving quality of life, enhancing walking speed and balance ability, promoting finger dexterity and reducing the fear of falls in patients with PD; 2) When the exercise period is ≤ 8 weeks or the total number of sessions is ≤ 30, home-based exercise may not be effective in relieving the overall motor symptoms of PD patients. These results can be used to prescribe home exercise for PD patients.

Numerous studies have demonstrated the deleterious effects of insufficient physical activity on motor symptoms in PD (e.g., decreased muscle strength, balance, and walking ability). As early as 2011, results from Nimwegen and colleagues showed that greater disease severity, gait impairment, and greater disability in daily living were associated with less daily physical activity in PD (R2 = 24%) [38]. The study results of Snider, et al. (2015) [39] also showed an inverse relationship between motor UPDRS severity scores and duration of non-exercise physical activity (*R* =  − 0.37, *P* = 0.0099). In the same year, a cross-sectional study by Bryant, et al. (2015) [40] showed that falls and fear of falling were associated with more ADL limitations and less physical activity. Improving muscle strength, balance, and walking ability is critical for alleviating the risk of falls or falls in PD [41, 42]. Findings from a recent review suggest that reducing sedentary behaviors (e.g., reducing television viewing time) and increasing physical activity may be effective in improving the quality of life of people with PD [43].

During the health crisis caused by the COVID-19 pandemic, physical inactivity has been further exacerbated by forced social isolation [44]. In fact, age is the biggest risk factor for exacerbating PD [45]. and the older adult have been identified as the age group most at risk of contracting COVID-19 [46]. which is why self-isolation measures are especially targeted at those over 65 [47]. In response to this unprecedented limitation of physical activity, home-based exercise seems to inevitably alleviate the physical inactivity of the older adults and improve and maintain the physical health, activities of daily living and independence of the elderly [48]. Our findings also suggest that home-based exercise appears to be effective in relieving overall motor symptoms, improving quality of life and functional performance (e.g., walking and balance, improving finger dexterity, and reducing fear of falls) in PD patients. Therefore, it is recommended that PD patients can choose home-based exercise to relieve motor symptoms and improve their quality of life during the COVID-19 pandemic.

Our results showed that both home-based aerobic exercise and mixed exercise were effective in reducing overall motor symptoms (as reflected on the UPDRS motor scale) in PD patients, even though the effects were small (SMD = -0.29 to -0.35 < 0.5). The previous meta-analyses indicated that supervised exercise, including aerobic exercises, strength training, and a combination of various exercise types, was an effective means to alleviate motor symptoms in PD patients [49–51]. Parcianello Cabeleira, et al. (2022) [52] conducted a meta-analysis combining four studies involving 261 PD patients. Their findings from the meta-analysis suggested that home-based minimally supervised exercise demonstrated similar effectiveness in alleviating motor symptoms in PD patients compared to professionally supervised exercise. Previous studies have shown that exercise can have beneficial effects on PD patients through neuroplasticity, protection of nerve cells from brain damage, and modulation of neurotrophic factors [3]. Among different types of exercise programs, aerobic exercise is considered the best option for improving an individual's lifelong health [53]. Supervised stationary bike aerobic exercise is not only safe, but also improves aerobic capacity, exercise performance and cognitive function in patients with early PD patients [54]. This is consistent with the results of a recent study published in The Lancet that remotely supervised home-based aerobic exercise effectively relieves motor symptoms in Parkinson's patients [11]. It is also consistent with the results of this study. Therefore, we recommend home-based aerobic exercise as the type of exercise during the COVID-19 pandemic.

For mixed exercise types (combination of aerobic, strength, and balance exercises), previous studies have shown that a combination of multimodal exercise types is more effective than a single exercise form in improving muscle strength and balance in older adults [55]. It is worth noting that the mixture of multimodal movement types, in terms of learning difficulty, may be challenging for PD patients with lower motor ability, because high motor skills are required to perform various movements. In contrast, a single training modality that only needs to focus on one training type allows PD patients to maintain relatively good exercise dosage and technical execution throughout their training program. This may partly explain why the home-based mixed exercise and single aerobic exercise were similar in improving the overall motor symptoms of Parkinson's patients in this study.

The results of this study suggest that home-based exercise may not be effective in relieving the overall motor symptoms of PD patients when the exercise cycle is ≤ 8 weeks or the total number of sessions is ≤ 30. A recent systematic review and meta-analysis showed no dose–response relationship between aerobic exercise dose (Frequency, period, number of sessions, and session duration) and overall motor symptoms in PD patients, unfortunately this study did not assess aerobic exercise minimum dose to relieve overall motor symptoms in PD patients [56]. This is the biggest highlight of this study, which provides clinicians and physical therapists with recommendations for minimum doses of home-based exercise prescriptions for PD patients, that is, the exercise cycle lasts for more than 8 weeks and the total number of courses exceeds 30 times. In addition, the results of this study showed that 8–16 weeks of home-based exercise achieved a moderate effect (SMD = -0.6 > 0.5) and the best effect in relieving the overall motor symptoms of PD patients. This is inconsistent with the findings of de Almeida, et al. (2022) [56] (higher aerobic exercise dosage (up to 64 weeks) presented higher effect sizes). On the one hand, long-term exercise (> 16 weeks) may be more likely to lead to a decline in the quality of home exercise in PD patients due to lack of supervision. In addition, the subgroup of included studies greater than 16 weeks had two studies with less than 70% course compliance, and two studies did not report course compliance. In the 8–16 weeks subgroup, compliance was as high as 95% in both included studies. Therefore, we have reason to believe that the low compliance rate of long-term home-based exercise may also lead to poor exercise benefits. In addition, more than 3 times a week and more than 120 min of exercise per week also showed a moderate effect. This gives us inspiration that, in the case that long-term home-based exercise cannot avoid a decrease in course quality and exercise dose due to lack of supervision, we can make up for the lack of long-term home-based exercise by increasing the frequency and time of weekly training. However, it is worth noting that the differences between all independent training factor analyses were not significant. Therefore, our findings must be interpreted with caution.

However, several limitations of the present review warrant mention. First, there is a possibility that studies meeting the inclusion criteria were not included in this meta-analysis, especially ongoing research and studies reporting negative findings. This bias may affect the reliability of our findings to some extent. In addition, the participants in the included study were not forced to isolate at home to exercise, so our results may not reflect the true impact of exercise on PD patients during forced home isolation. Due to differences in the units or scales of the included outcomes (e.g., motor symptoms include UPDRS-III and MDS-UPDRS-III), we chose SMD values for the pooled effect size, which made our results unable to assess minimal clinically important difference. Therefore, it is unknown whether the results of the study achieve clinical significance.

## Conclusion

In conclusion, home-based exercise appears to be effective in relieving motor symptoms and improving quality of life in PD patients. Therefore, during times of limited physical activity caused by pandemics such as COVID-19, or have limited mobility, preventing them from participating in supervised exercise programs, home-based exercise is an alternative to maintain and improve the health of PD patients. In addition, for the minimum dose of home-based exercise, we recommend that the exercise period is no less than 8 weeks and the total number of sessions is no less than 30 times. This provides clinicians and patients with clear evidence-based clinical practice.

## Data Availability

All data generated or analysed during this study are included in this published article [and its supplementary information files].

## Abbreviations

BBS: Berg balance scale
CI: Confidence intervals
COVID-19: Coronavirus disease 2019
FES-I: Fall efficacy scale–international questionnaire
GGOY: Guoguang Ouyang
HYZ: Haoyang Zhang
PD: Parkinson disease
PDQ-39: The Parkinson's Disease Questionnaire
PEDro: Physiotherapy Evidence Database scale
PRISMA: The Preferred Reporting Items for Systematic Reviews and Meta-Analyses
SCL: Shu-Cheng Lin
SD: Standard deviation
SEM: Standard error of the mean
SMD: Standardised mean difference
UPDRS-motor: Unified Parkinson's Disease Rating Scale-motor symptoms
WHO: World Health Organization
XYF: Xueying Fu
YY: Yong Yang
